# Spatio-temporal evolution of land use and its eco-environmental effects in the Caohai National Nature Reserve of China

**DOI:** 10.1038/s41598-023-47471-4

**Published:** 2023-11-17

**Authors:** Yin Su, Guojun Feng, Jintong Ren

**Affiliations:** 1 College of Eco-Environmental Engineering, Guizhou Minzu University, Huaxi Dist, Guiyang , 550025 Guizhou China; 2 Guizhou Province Key Laboratory of Ecological Protection and Restoration of Typical Plateau Wetlands, Guizhou University of Engineering Science, Bijie, 551700 Guizhou China

**Keywords:** Wetlands ecology, Environmental impact

## Abstract

With the rapid development of social economy, the ecological environment problems caused by the change of wetland land use have been widely concerned. This paper takes the Caohai National Nature Reserve (CNNR) of China as the research object on the basis of referring to previous research results. Firstly, the remote sensing data was employed to examine the spatio-temporal evolution process of the CNNR from three aspects: land use structure change, land use dynamic degree and land use space change. Then the change of ecological environment quality was studied from the greenness, the wetness, the dryness and the heat. Based on the spatiotemporal changes of land use types and ecological environment quality in the CNNR from 2000 to 2020, a comprehensive index, the remote sensing ecological index (RSEI), was constructed to analyze the ecological environmental effects of land use changes. The results indicate that the land use changes in the CNNR went through two major periods: first, a period of rapid decline in cultivated land, and second, a period of sharp increase in constructed land. During the period of rapid decline in cultivated land, the ecological environment quality in the study area showed an upward trend. However, during the period of increased constructed land, the ecological environment quality gradually stabilized. This study provides a basis for the coordinated development of the ecological environment and social economy in the CNNR area.

## Introduction

Land use is the process through which human activities intervene in the natural and economic reproduction of land, and the ecological environment encompasses the sum of various ecological factors and relationships upon which organic life relies for development, survival, reproduction, and evolution^1^. In recent years, with the development of the economy and society, the expansion of urban areas, there have been fundamental changes in the types, structures, and patterns of land use, leading to a series of ecological environmental issues such as air pollution, soil degradation, intensified urban heat island effect, and biodiversity loss^2–4^. Land use change is considered the most significant driving force behind ecological environmental changes, so it is crucial to understand the ecological and environmental impacts of various land types. Each land type exerts either a positive or negative influence on the quality of the ecological environment. Forested areas, in general, are characterized by predominantly positive effects^5^. They play a pivotal role in water retention, erosion control, soil structure enhancement, organic matter accumulation, and the improvement of air quality. Forested regions boast rich biological resources and represent the most diverse terrestrial ecosystems in terms of biodiversity. Grasslands also contribute primarily positive environmental impacts, albeit to a lesser extent than forested land. Cultivated land and orchards are designated for crop and fruit production, and their environmental impact varies based on farming practices and the application of fertilizers. Fertilizer usage significantly contributes to the eutrophication of rural water bodies, accounting for up to 50% of such cases^6^. These lands simultaneously meet human food requirements while serving ecological functions such as carbon absorption and oxygen production. The implementation of sustainable agricultural practices can further amplify these positive ecological impacts and diminish associated negative effects. Construction land imposes detrimental environmental consequences. The rapid expansion of construction land has emerged as a significant driver of ecological degradation. The substantial increase in the area of construction land often results in the loss of extensive agricultural land, particularly high-quality cultivated land^7^. Furthermore, it leads to the release of pollutants, causing varying degrees of environmental contamination in the atmosphere, water bodies, and soils, ultimately disrupting the ecological equilibrium. Ecological land primarily encompasses water bodies, wetlands, forests, and natural reserves. These areas serve as critical ecological functional zones, exhibiting clear-cut positive effects on the environment. They play a pivotal role in water retention, erosion mitigation, biodiversity enhancement, and hold significant value in terms of natural resources and cultural landscapes. Therefore, investigating the mechanisms by which land use affects the ecological environment is a fundamental scientific question that will contribute to China's ecological civilization construction and rural revitalization. Additionally, it will promote interdisciplinary development in disciplines such as land resource science, ecology, and environmental science.

Land use and land cover change (LUCC) research has become a prominent focus in the study of global ecological environmental changes^8,9^. The earliest LUCC research program was jointly proposed by the International Geosphere-Biosphere Program (IGBP) and the Human Dimensions of Global Environmental Change Program (IHDP) in 1995^10^. Previous studies on LUCC primarily focused on two aspects. Firstly, from the perspective of landscape ecology, researchers explored the mechanisms of land use change’s impact on ecological processes, changes in landscape patterns, and the influence of landscape indices^11–13^. Secondly, by employing methods such as land use transfer matrix, ecological environment quality index, and land use transition ecological contribution rate, researchers analyzed the ecological environmental effects of land use change^14–16^. Existing literature mainly investigates the ecological and environmental impacts of land use change based on land use classification and the spatiotemporal evolution of land use^17,18^. These studies encompass various aspects, including atmospheric environment^19^, water environment^20^, soil environment^21^, biological environment^22^, and ecosystem services^23,24^. In recent years, there has been a growing interest among scholars in the comprehensive assessment of ecological environment quality by combining multiple individual indicators. One notable example is the environmental quality index (EQI)^25^, which examines the correlation between environmental quality and human health. Another noteworthy index is the ecological index (EI), which integrates six distinct indicators^26^, widely adopted by the Chinese government as the primary tool for evaluating ecological environment quality. Additionally, the environmental stress index (ESI) serves to assess the impact of external disturbances on the local environment^27^. Meanwhile, the energy security and environmental sustainability index (ESESI) offers an evaluation of both energy security and environmental sustainability^28^. These emerging indices are designed to consider factors such as data availability and the scientific rigor of environmental assessments, reflecting a more holistic approach to evaluating ecological environment quality. In the early twenty-first century, Chinese scholars introduced land use transition theory into LUCC-related research, which subsequently received sustained attention domestically^29^. Numerous studies have explored the ecological and environmental responses to land use change^30^. However, due to the complexity of the processes and mechanisms by which land use change impacts the ecological environment, future research trends are likely to focus on interdisciplinary integration, the fusion of “3S” technology and simulation models, and the exploration of feedback mechanisms of ecological environmental responses.

The Caohai National Nature Reserve (CNNR) of China belongs to the typical karst plateau wetland type. It is located in the water source area of the Yungui Plateau and plays a crucial role in maintaining the regional ecosystem balance and ensuring downstream water supply. However, in recent years, improper land development and environmental pollution have severely damaged the structure and function of the wetland ecosystem. The wetland area has shrunk, posing a threat to local socioeconomic development. There is a lack of intuitive understanding regarding the feedback relationship between land use and the ecological environment, and the strategies for balanced development are lacking scientific guidance. Current research on the Caohai Wetland in the karst plateau primarily focuses on biodiversity^31^, water quality and hydro-environment^32^, and the assessment of ecosystem service value^33^. However, there is limited research on the coupled feedback relationship between land use and the ecological environment of the CNNR and their balanced development under the dual disturbances of natural changes and human activities. Therefore, it is urgently necessary to have a clear understanding of the following scientific questions: What are the spatiotemporal characteristics of land use changes in the CNNR? What are the spatiotemporal differences in the ecological environmental effects caused by land use changes? How can they achieve sustainable development through their interactions?

In conclusion, this study focuses on the CNNR, a karst plateau lake wetland, and collects long-term time series remote sensing monitoring imagery data of land use in the CNNR Wetland. By utilizing the remote sensing ecological environment index (RSEI) measurement method, the study investigates the spatiotemporal evolution characteristics of land use changes and the ecological environmental responses in the CNNR Wetland. The findings aim to provide scientific evidence for the management, conservation, and sustainable development of the CNNR Wetland ecosystem. The purpose of this study was as follows: (1) to clarify the spatio-temporal pattern of land use change in the CNNR; (2) to determine the eco-environmental effects of land use transition by RSEI in the CNNR; and (3) to discuss the eco-environmental impact mechanism of land use change in the CNNR. The research findings may provide a scientific basis and policy enlightenment for addressing the conflict between land use development and environment protection, optimizing the allocation of land use resources and promoting the improvement of ecological environment quality in karst plateau wetland areas.

## Materials and methods

### Study area and data sources

**Figure 1:**
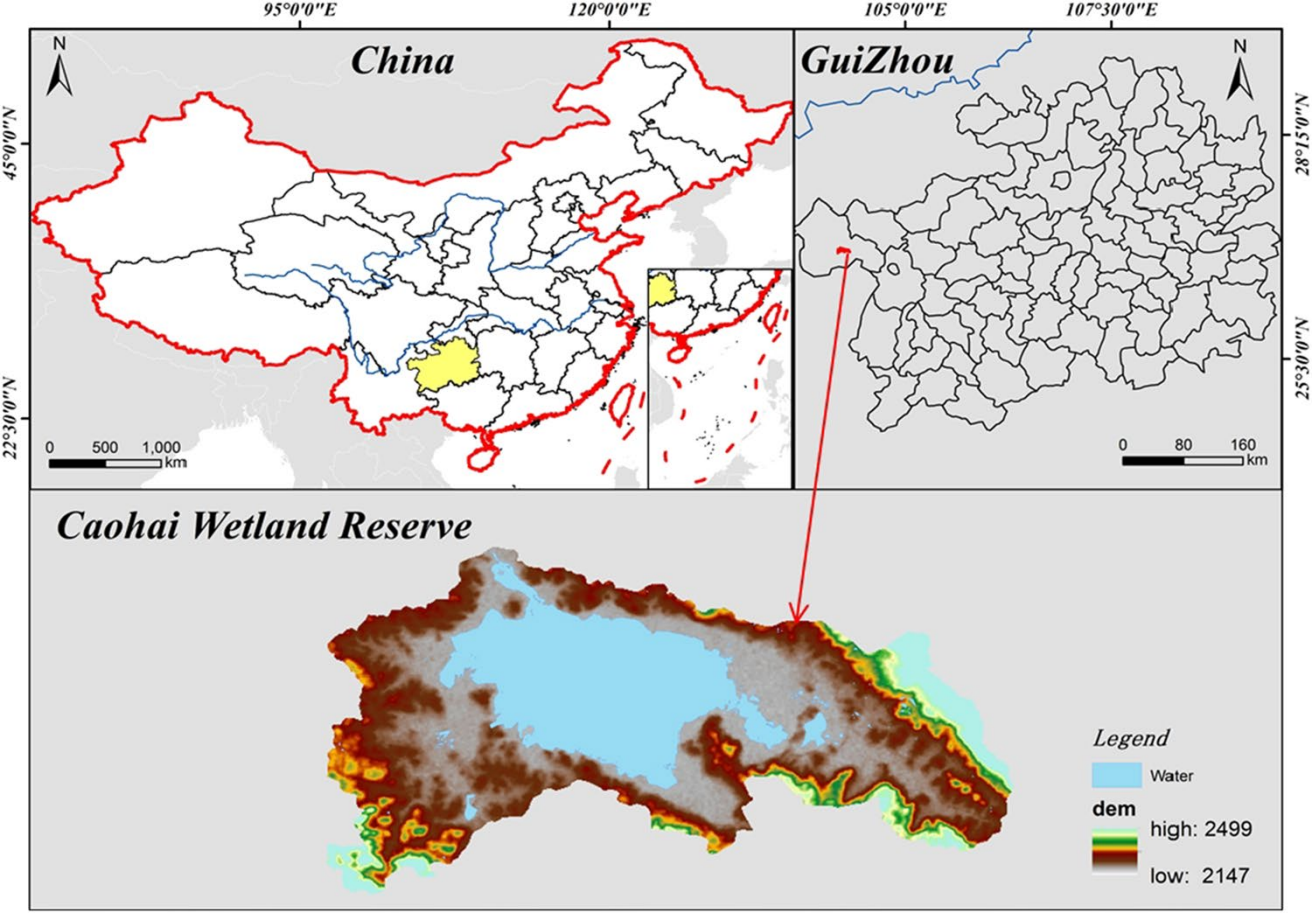
Overview of the study area. The figure is created using ArcMap 10.7, https://www.arcgis.com.

The Guizhou Caohai National Nature Reserve is located in the hinterland of the Wumeng Mountains, at the northwest edge of Guizhou Province, southwest of the county town of Weining Autonomous County. It is situated between 26° 47′ 32″ to 26° 52′ 52″ N latitude and 104° 10′ 16″ to 104° 20′ 40″ E longitude, covering a total area of 9600 ha (Fig. 1). The reserve falls within the subtropical plateau monsoon climate zone, characterized by abundant sunshine, mild winters, cool summers, and a distinct dry season and wet season. The average annual temperature is 10.5 °C, with an average annual precipitation of 950.9 mm and an average relative humidity of 80%. The area enjoys excellent solar energy resources, with an average annual sunshine duration of 1805.4 h. Caohai, the largest natural freshwater lake on the Guizhou Plateau, comprises deepwater areas, shallow marshes, sedge wetlands, meadows, and diverse aquatic flora and fauna. It possesses a well-structured and functional wetland ecosystem with high productivity, making it a representative example of subtropical plateau wetland ecosystems in China.

The remote sensing images, digital elevation models (DEMs), and other foundational data were sourced from the Resource and Environment Science Data Center (RESDC) of the Chinese Academy of Sciences, available at http://www.resdc.cn, as well as the Geospatial Data Cloud (http://www.gscloud.cn). The remote sensing image data used in this study for the years 2000 and 2010 were obtained from NASA’s Landsat TM satellite, while the data for 2020 were acquired from Landsat 8, with a spatial resolution of 30 m. The three sets of image data underwent processes such as band synthesis, geometric correction, and image enhancement. They were interpreted through interactive human–machine interaction, achieving an interpretation accuracy of 89.3%, and the Kappa coefficient was all above 0.8, meeting the accuracy requirements for remote sensing interpretation. Following the 3-level classification system of China’s land use/land cover data, the land use types in the study area were reclassified into six categories: cultivated land, forestland, grassland, water bodies, construction areas, and unused land.

### Methods

#### Land use transfer analysis

The land use changes in the CNNR from 2000 to 2020 are analyzed in terms of structural changes, dynamic degree changes, and spatial changes. This analysis aims to establish a foundation for studying the eco-environmental effects of land use changes. The focus of land use structural evolution is to examine the structural changes of various land classes during specific time periods. The evolution of land use dynamic degree is measured by the single land use dynamic degree and the comprehensive land use dynamic degree indicators. The spatial evolution of land use is reflected through land use transfer matrices and spatial visualization, which indicate the transfer directions of different land classes.

##### Change of land use structure

Land use structural change refers to the changes in the areas of different types of land in a certain region during a certain period of time. It mainly includes changes in the proportion of different land uses and the structural changes of land use types. Land use structural change can reflect the influence of various factors such as regional economic development, social changes, and environmental changes. It is of great significance for formulating rational land use policies, protecting the ecological environment, and promoting sustainable development. Usually, remote sensing technology and GIS technology are used to monitor and analyze land use structures^34^. The following is the formula:

1 $$K = Q_{b} - Q_{a} ,$$

where, *K* is the change rate of land use structure; *Q*_*b*_ is the proportion of a certain land use type area in year *b*; *Q*_*a*_ is the proportion of a certain land use type area in year *a*.

##### Land use dynamic degree

The dynamic degree of land use types is an index which is used to measure the extent of land use changes caused by human activities in different time periods^35,36^.

*The single index of land use dynamics* The indicator is the proportion of the area change of a certain land use type during a certain period of time to its initial area. The higher the land use dynamic degree, the greater the area change of the land use type during the period^37^. Its calculation formula is:

2 $$K_{1} = \frac{{U_{b} - U_{a} }}{{U_{a} }} \times \frac{1}{b - a},$$

where, *K*_1_ is the single land use dynamic degree; *U*_*a*_ represents the area of a certain land use type in year *a*; *U*_*b*_ represents the area of a certain land use type in year *b*.

*The comprehensive index of land use dynamics* The comprehensive index of land use dynamics divides land use types into several levels according to the degree of human impact on land and assigns different values to different land types. It can reflect both the natural attributes of land and the results of the interaction between human activities and the natural environment. The higher the grading index, the greater the impact of social and economic activities on the land. In this index, unused land is assigned a value of 1, forest land and water bodies are assigned a value of 2, cultivated land is assigned a value of 3, and construction land is assigned a value of 4. The calculation method is shown below^38^:

3 $$K_{2} = 100 \times \sum\limits_{n = 1}^{m} {A_{n} \times C_{n} } ,$$

where, *K*_2_ is the comprehensive index of land use dynamics, *K*_2_ ∈ [100, 400]; *A*_*n*_ is grading index of the *n*th land use degree; *C*_*n*_ is percentage of the area of the *n*th land use degree; *m* is number of land use degree levels.

The comprehensive change value of land use dynamics ∆I can quantitatively analyze the comprehensive level and trend of land use in the region, and measure the degree of joint action of various land types in a certain period of time. The calculation method is shown in formula (4). When ∆I is less than 0, it indicates that the land use in the region is in a declining period, and vice versa indicates a developing period.

4 $$\Delta I = K_{2b} - K_{2a} = 100 \times \left[ {\left( {\sum\limits_{n = 1}^{m} {A_{n} \times C_{nb} } } \right) - \left( {\sum\limits_{n = 1}^{m} {A_{n} \times C_{na} } } \right)} \right],$$

where, *C*_*na*_ represents the percentage of the area with the* n*th level of land use degree at time point *a*; *C*_*nb*_ represents the percentage of the area with the *n*th level of land use degree at time point *b*.

##### Spatial change of land use

The land use transfer matrix is used to analyze the spatial changes of land use types by calculating the area and proportion of land use types in different periods. The transfer matrix shows the direction and magnitude of changes in land use types, reflecting the trend of land use conversion. The land use transfer spatial map is a visual representation of the land use changes in different periods. By comparing the land use transfer spatial maps of different periods, we can visually identify the changes in land use patterns, the expansion or contraction of different land use types, and the spatial distribution of land use changes. By using the land use transfer matrix and spatial map analysis methods, this study provides a comprehensive understanding of the land use changes in the study area, which can be used to guide land use planning and management, and to protect the ecological environment.

#### Eco-environmental quality evaluation

Existing literature have verified the effectiveness of RSEI in evaluating the ecological environment^39,40^. The index incorporates four indicators: wetness (WET), greenness (NDVI), dryness (NDBSI), and heat (LST). By considering elements associated with human well-being and habitation, such as vegetation, temperature, and soil, this index offers a comprehensive assessment of the ecological environment status of the region.

##### Greenness indicator (NDVI)

The normalized difference vegetation index (NDVI) is a widely used vegetation index that can effectively reflect the status and spatial distribution of plant growth. It can represent the greenness indicator in this paper, and the calculation formula is as follows^41^:

5 $$NDVI = \left( {NIR - R} \right)/\left( {NIR + R} \right),$$

where, *NIR* and *R* refer to the reflectance of the near-infrared and red bands, respectively.

##### Wetness indicator (WET)

The wetness indicator uses the wetness component obtained through Tasseled Cap Transformation to measure the humidity status of vegetation and soil in the region. Generally speaking, the humidity in vegetation-covered areas is higher than that in non-vegetation-covered areas (excluding water bodies). The calculation methods for Wet in TM data and OLI/TIRS data are shown in the following formulars^42^.

TM data,

6 $$Wet{ = }\left( \begin{aligned} 0.0315\rho_{Blue} \, &+ \, 0.2021\rho_{Green} + 0.3102\rho_{Red} + 0.1594\rho_{NIR} \hfill \\ &- \;0.6806\rho_{SWIR1} - \;0.6109\rho_{SWIR2} \hfill \\ \end{aligned} \right).$$

OLI/TIRS data,

7 $$Wet{ = }\left( \begin{aligned} 0.1511\rho_{Blue} \, &+ \, 0.1973\rho_{Green} + 0.3283\rho_{Red} + 0.3407\rho_{NIR} \hfill \\ &- \;0.7171\rho_{SWIR1} - \;0.4559\rho_{SWIR2} \hfill \\ \end{aligned} \right),$$

where, *ρ*_*Blue*_,* ρ*_*Green*_,* ρ*_*Red*_, *ρ*_*NIR*_,* ρ*_*SWIR1*_, *ρ*_*SWIR2*_ represent the reflectance of the blue, green, red, near-infrared, shortwave infrared 1, and shortwave infrared 2 bands, respectively.

##### Dryness indicator (NDBSI)

The dryness index can characterize the land degradation and surface bareness. In this study, it was used to represent the dryness index by combining the soil index (SI) and the built-up index (IBI), which can reflect the dryness condition of the study area. The calculation formula is as follows^43^:

8 $$SI{ = }\frac{{\left( {\rho_{SWIR1} + \rho_{Red} } \right) - \left( {\rho_{NIR} + \rho_{Blue \, } } \right)}}{{\left( {\rho_{SWIR1} + \rho_{Red} } \right) + \left( {\rho_{NIR} + \rho_{Blue} } \right)}},$$

9 $$IBI{ = }\left[ {\frac{{2\rho_{SWIR1} }}{{\rho_{SWIR1} + \rho_{NIR} }} - \left( {\frac{{\rho_{NIR} }}{{\rho_{NIR} + \rho_{Red} }} + \frac{{\rho_{Green} }}{{\rho_{Green} + \rho_{SWIR1} }}} \right)} \right]/\left[ {\frac{{2\rho_{SWIR1} }}{{\rho_{SWIR1} + \rho_{NIR} }} + \left( {\frac{{\rho_{NIR} }}{{\rho_{NIR} + \rho_{Red} }} + \frac{{\rho_{Green} }}{{\rho_{Green} + \rho_{SWIR1} }}} \right)} \right],$$

10 $$NDBSI = (SI + IBI)/2.$$

##### Heat indicator (LST)

The heat index is characterized by the surface temperature inverted from the thermal infrared band of remote sensing images. The greater the value of the heat index, the higher the surface temperature. The calculation formula is as follows^44^:

11 $$L_{{6/10}} = g_{ain} \times N_{D} + b_{ias} ,$$

12 $$B_{LST} = \left[ {L_{{6/10}} - L_{1} - \tau \left( {1 - \varepsilon_{{6/10}} } \right)L_{2} } \right]/\tau \times \varepsilon_{{6/10}} {,}$$

13 $$LST = K_{2} /\ln (K_{1} /B_{LST} + 1) - 273,$$

where *L*_6/10_ represents the radiation value of the thermal infrared band. The thermal infrared bands of different sensors are different. The thermal infrared band of Landsat 5 TM image data is the 6th band, while the thermal infrared band of Landsat 8 OLI remote sensing image is the 10th band. *N*_*D*_ represents the pixel grayscale value, *g*_*ain*_ represents the gain value of the thermal infrared band, and *b*_*ias*_ is the offset value of the thermal infrared band. *K*_1_ and *K*_2_ represent the calibration parameters of the sensor. *L*_1_ represents the upward radiance of the atmosphere, while *L*_2_ represents the downward radiance of the atmosphere. *τ* represents the transmittance of the atmosphere in the thermal infrared band of the remote sensing image, and *ε*_6/10_ represents the surface emissivity. *B*_*LST*_ represents the blackbody radiance value. The values of *L*_1_, *L*_2_, and *τ* can be obtained from the official atmospheric parameter query website (https://atmcorr.gsfc.nasa.gov/) of the National Aeronautics and Space Administration (NASA).

##### Constructing the RSEI

The RSEI integrates four indicator factors, namely Greenness (NDVI), Wetness (WET), Dryness (NDBSI), and Heat (LST), to assess the ecological condition of a given region. After calculating the four indicator components from remote sensing imagery, each component is individually subjected to standardization. The standardized values of the four indicators are then combined using the band synthesis tool in ENVI 5.3 software. Subsequently, Principal Component Analysis (PCA) is employed to calculate the RSEI for the study area.

In the process of PCA, directly calculating PCA on four indices with inconsistent units would result in an imbalance of weights among the indices. Therefore, it is necessary to standardize the four indices first, unifying their units within the range of 0 to 1. Subsequently, PCA is performed, and the first principal component band (PC1) in the PCA output represents the initial value of the remote sensing ecological index (RSEI0) sought after. The RSEI is then generated by directly standardizing PC1. The formula is as follows^45^:

14 $$RESI = (PC1 - PC1_{\min } )/(PC1_{\max } - PC1_{\min } ),$$

where *PC1*_min_ represents the minimum value of the first principal component *PC*1, and *PC1*_max_ represents the maximum value of *PC*1.The *RSEI* in the equation refers to the constructed remote sensing ecological index, where a higher value indicates a better ecological quality, while a lower value indicates a poorer ecological quality.

### Research consent statement

This study was approved by Caohai National Nature Reserve Administration Committee.

## Results

### Land use transfer analysis

#### Change of land use structure

The entire Caohai Nature Reserve is divided into three distinct regions for analysis, namely the Southwest Zone, the Caohai Lake Perimeter Zone, and the Eastern Zone. The Southwest Zone predominantly consists of grassland, with pockets of forested and cultivated land interspersed, alongside a small portion of unused land. Notably, there has been a gradual increase in construction land starting from the year 2010.

The Caohai Lake Perimeter Zone is primarily characterized by grasslands and cultivated land, with some areas of forestland situated in the eastern part of the region. Between the years 2000 and 2010, there was a marked expansion of cultivated land, accompanied by a reduction in forested areas. Subsequently, from 2010 to 2020, there was a significant decrease in cultivated land and an increase in forestland coverage.

**Figure 2:**
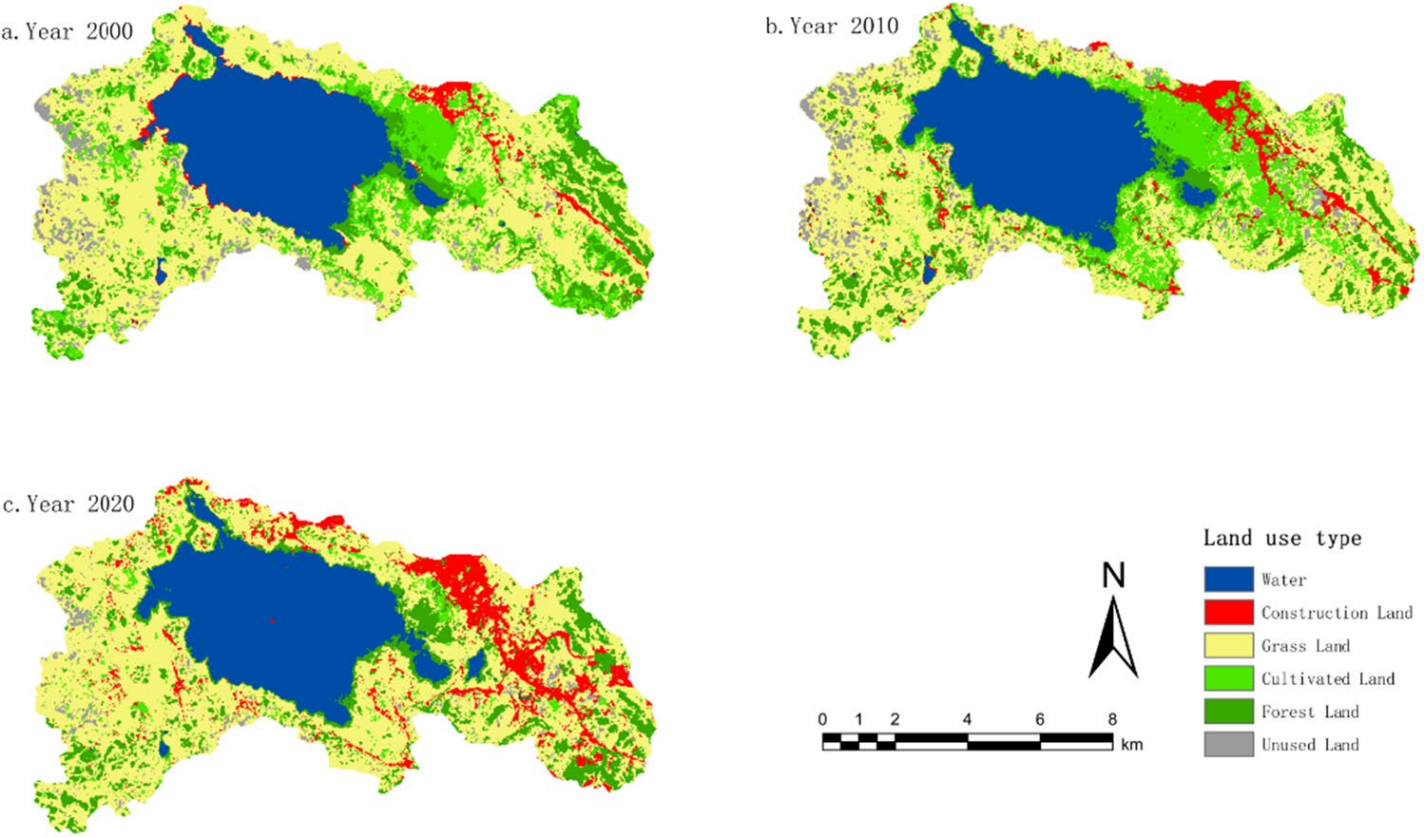
Land use spatial changes from 2000 to 2020. The figure is created using ArcMap 10.7, https://www.arcgis.com.

The Eastern Zone, in the year 2000, was primarily characterized by grassland and cultivated land. However, by 2010, cultivated land had become the dominant land type, and by 2020, it had evolved into the predominant category of land use, with urban development significantly overshadowing cultivated land, resulting in a rapid decline in cultivated land coverage (Fig. 2).

At the same time, the Table 1 reflects the trends in land use structure changes in the CNNR over different periods. From an encompassing perspective of the entire CNNR, compared to the period of 2000–2010, the rate of land use structure changes during 2010–2020 was relatively higher. In terms of water bodies, the overall change was minimal, showing a slight increase. Over the 20-year period, the water area increased by 1.494 km^2^. The land allocated for buildings within the reserve increased from 2.313 km^2^ in 2000 to 9.034 km^2^ in 2020, indicating a rapid increase trend. Over the 20-year period, the building land increased by 6.721 km^2^. The grassland area decreased from 45.484 km^2^ in 2000 to 40.613 km^2^ in 2010 but then increased to 48.417 km^2^ in 2020. This demonstrates a pattern of initial decrease followed by an increase. Over the 20-year period, the grassland area increased by 2.933 km^2^. The cultivated land area experienced the greatest change among all land categories. In the first 10 years, it showed a slow increasing trend, rising from 13.487 km^2^ in 2000 to 14.747 km^2^ in 2010. However, in the subsequent 10 years, due to the impact of land conversion policies, the cultivated land area rapidly decreased, with a total reduction of 12.29% during that period. The forested area showed minimal overall change, exhibiting a pattern of initial decrease followed by an increase. It increased from the initial 12.152 km^2^ to 13.773 km^2^. The area of unused land initially increased and then decreased, with a total reduction of 1.832 km^2^ over the 20-year period.

**Table 1:** Area and change rate of land use types in CNNR from 2000 to 2020.

Land use types	Area (km^2^)			Change rate* K* (%)		
	2000	2010	2020	2000–2010 (%)	2010–2020 (%)	2000–2020 (%)
Water	21.974	23.027	23.468	1.06	0.44	1.51
Construction land	2.313	4.568	9.034	2.27	4.50	6.77
Grassland	45.484	40.613	48.417	− 4.91	7.86	2.96
Cultivated land	13.487	14.747	2.551	1.27	− 12.29	− 11.02
Forest land	12.152	12.094	13.773	− 0.06	1.69	1.63
Unused land	3.848	4.210	2.016	0.36	− 2.21	− 1.85

#### Change of land use dynamics

Based on Table 2, the analysis of single land use change and annual variation rate in the CNNR during different periods is as follows.

**Table 2:** The change of single land use dynamics in the CNNR from 2000 to 2020 (km^2^).

Land use types	2000–2010		2010–2020		2000–2020	
	Change value	Annual variation rate *k*_*2*_ (%)	Change value (km^2^)	Annual variation rate *k*_*2*_ (%)	Change value (km^2^)	Annual variation rate *k*_*2*_ (%)
Water	1.053	1.20	0.441	0.48	1.494	1.70
Construction land	2.255	24.37	4.467	24.45	6.721	72.65
Grassland	− 4.871	− 2.68	7.8039	4.80	2.933	1.61
Cultivated land	1.259	2.33	− 12.196	− 20.68	− 10.937	− 20.27
Forest land	− 0.058	− 0.12	1.679	3.47	1.621	3.33
Unused land	0.362	2.35	− 2.194	− 13.03	− 1.832	− 11.90

From 2000 to 2010, the water area in the CNNR increased at an average rate of 1.02% per year, with a total increase of 1.053 km^2^. The area of land allocated for buildings had the highest variation rate among all land categories due to its smaller base, showing a significant annual increase rate of 24.37%. Over the 10-year period, the building land area increased by 2.255 km^2^. The grassland area experienced the largest change, decreasing at an average rate of 2.33% per year, resulting in a total decrease of 4.871 km^2^. The cultivated land area increased at an average rate of 2.33% per year, with a total increase of 1.259 km^2^. The forested area had minimal variation, decreasing at an average rate of 0.12% per year by a small amount of 0.058 km^2^. The area of unused land increased at an average rate of 2.35% per year, resulting in a total increase of 0.362 km^2^. Based on the magnitude of the annual variation rates, the land use changes in the CNNR can be ranked as follows: building land (24.37%) > grassland (-2.6%) > unused land (2.35%) > cultivated land (2.33%) > water bodies (1.20%) > forested land (-0.12%).

From 2010 to 2020, the change in water area was minimal, increasing at an average rate of 0.48% per year, with a total increase of 0.441 km^2^ over the 10-year period. The area of land allocated for buildings continued to experience high-speed growth, with an annual average growth rate of 24.45% and a substantial increase of 4.467 km^2^ over the 10-year period. The grassland area exhibited a rapid increase, growing at an average rate of 4.80% per year and increasing by 7.8039 km^2^ over the 10-year period. Among all land categories, the cultivated land area experienced the largest change, decreasing at an average rate of 20.68% per year and resulting in a total decrease of 12.196 km^2^. The forested area increased by 3.47% per year, with a total increase of 1.679 km^2^. The area of unused land decreased at an average rate of 13.03% per year, resulting in a total reduction of 2.194 km^2^. Based on the magnitude of the annual variation rates, the land use changes in the CNNR can be ranked as follows: building land (24.45%) > cultivated land (− 20.68%) > unused land (− 13.03%) > grassland (4.80%) > forested land (3.47%) > water bodies (0.48%).

From 2000 to 2020, the water area showed minimal overall change, increasing at an average rate of 1.70% per year and a total increase of 1.494 km^2^ over the 20-year period. The area of land allocated for buildings experienced the largest increase among all land categories, with a significant growth rate of 72.65% and a total increase of 6.721 km^2^. The grassland area showed a pattern of initial decrease followed by an increase, growing at an average rate of 1.61% per year and resulting in a total increase of 2.933 km^2^. The cultivated land area exhibited a pattern of initial increase followed by a decrease, with a more significant variation, leading to a total decrease of 10.937 km^2^ over the 20-year period. The forested area showed a pattern of initial decrease followed by an increase, with a relatively stable variation, resulting in a total increase of 1.621 km^2^. The area of unused land showed a pattern of initial increase followed by a decrease. However, due to its smaller base, the overall variation was substantial, with a total reduction of 1.832 km^2^ over the 20-year period. Based on the magnitude of the annual variation rates, the land use changes in the CNNR can be ranked as follows: building land (72.65%) > cultivated land (− 20.27%) > unused land (− 11.90%) > forested land (3.33%) > water bodies (1.70%) > grassland (1.61%).

According to formulas (3) and (4), the comprehensive land use dynamics index (*k*_2_) for the CNNR in the years 2000, 2010, and 2020 were calculated as 214.37, 218.82, and 219.74, respectively. Overall, there is an upward trend in the k_2_ values. From 2000 to 2020, the area of land allocated for buildings expanded rapidly in the CNNR. The cultivated land area slowly increased before 2010 but rapidly decreased after 2010. However, the k_2_ values were not high. This is due to the large base and proportion of water bodies, grassland, and forested land, as well as the influence of relevant policies within the protected area.

The comprehensive change values (∆I) of land use dynamics were positive from 2000 to 2020. The ∆I value for the period of 2000–2010 (4.45) was higher than the ∆I value for the period of 2010–2020 (0.92). This indicates that the land use in the CNNR experienced a rapid development phase during the 20-year period from 2000 to 2020. In comparison to the period of 2010–2020, the socio-economic activities and land use policies had a greater impact on land use during the period of 2000–2010.

##### Spatial change of land use

According to Table 3, during the period of 2000–2010, there were interchanges among various land types, with the largest conversion occurring between grassland and cultivated land. In terms of area increase, grassland had the highest increase, followed by cultivated land, with contribution rates of 32.95% and 30.62%, respectively. In terms of area decrease, grassland had the largest conversion out, contributing to a reduction rate of 46.48%. Compared with 2010–2020, grassland still has the largest increase, with a conversion rate of 49.44%. The decrease in cultivated land was the most significant, with a conversion rate of 42.55%, mainly converted into grassland and construction land (Table 4). Looking at the entire period from 2000 to 2020 (Table 5), the water area in the study area exhibited a stable growth trend, increasing by a total of 1.494 km^2^ over 20 years. The construction land saw a significant increase, growing by 290.58%. There was a tendency for the construction land to expand towards the core conservation area of the CNNR. The grassland showed significant fluctuations, with both increases and decreases. The cultivated land rapidly decreased. Forested land increased by 13.33%, while unused land decreased by 47.61%.

**Table 3:** Land use transfer matrix of CNNR from 2000 to 2010 (Unit: km^2^).

Land use types	Water	Construction land	Grassland	Cultivated land	Forest land	Unused land	Reduce area	2000 total area
Water	21.421	0.007	0.000	0.164	0.382	0.000	0.553	21.974
Construction land	0.472	1.574	0.068	0.064	0.133	0.002	0.739	2.313
Grassland	0.541	2.286	29.890	8.017	2.524	2.227	15.594	45.484
Cultivated land	0.021	0.321	6.378	4.780	1.866	0.122	8.708	13.487
Forest land	0.572	0.370	2.335	1.679	7.190	0.006	4.962	12.152
Unused land	0.000	0.009	1.942	0.043	0.000	1.854	1.994	3.848
Add area	1.606	2.993	10.724	9.967	4.904	2.356	32.549	
2010 total area	23.027	4.568	40.613	14.747	12.094	4.210		99.258

**Table 4:** Land use transfer matrix of CNNR from 2010 to 2020 (Unit: km^2^).

Land use types	Water	Construction land	Grassland	Cultivated land	Forest land	Unused land	Reduce area	2010 total area
Water	22.209	0.023	0.068	0.001	0.726	0.000	0.817	23.027
Construction land	0.002	3.058	1.252	0.014	0.189	0.053	1.509	4.568
Grassland	0.096	2.559	32.284	1.556	3.286	0.833	8.330	40.613
Cultivated land	0.443	2.388	8.609	0.864	2.317	0.127	13.883	14.747
Forest land	0.714	0.651	3.452	0.050	7.219	0.008	4.875	12.094
Unused land	0.004	0.356	2.753	0.066	0.036	0.995	3.215	4.210
Add area	1.258	5.976	16.133	1.687	6.554	1.021	32.629	
2020 total area	23.468	9.034	48.417	2.551	13.773	2.016		99.258

**Table 5:** Land use transfer matrix of CNNR from 2000 to 2020 (Unit: km^2^).

Land use types	Water	Construction land	Grassland	Cultivated land	Forest land	Unused land	Reduce area	2000 total area
Water	21.614	0.013	0.033	0.001	0.313	0.000	0.360	21.974
Construction land	0.356	1.247	0.404	0.006	0.282	0.017	1.066	2.313
Grassland	0.376	5.129	33.732	1.715	3.623	0.909	11.752	45.484
Cultivated land	0.242	1.396	8.265	0.711	2.732	0.141	12.776	13.487
Forest land	0.879	1.189	3.295	0.020	6.755	0.014	5.397	12.152
Unused land	0.000	0.060	2.688	0.097	0.068	0.934	2.914	3.848
Add area	1.854	7.787	14.685	1.840	7.018	1.082	34.2657	
2020 total area	23.468	9.034	48.417	2.551	13.773	2.016		99.258

### Eco-environmental quality evaluation

#### Greenness indicator change characteristics (NDVI)

**Figure 3:**
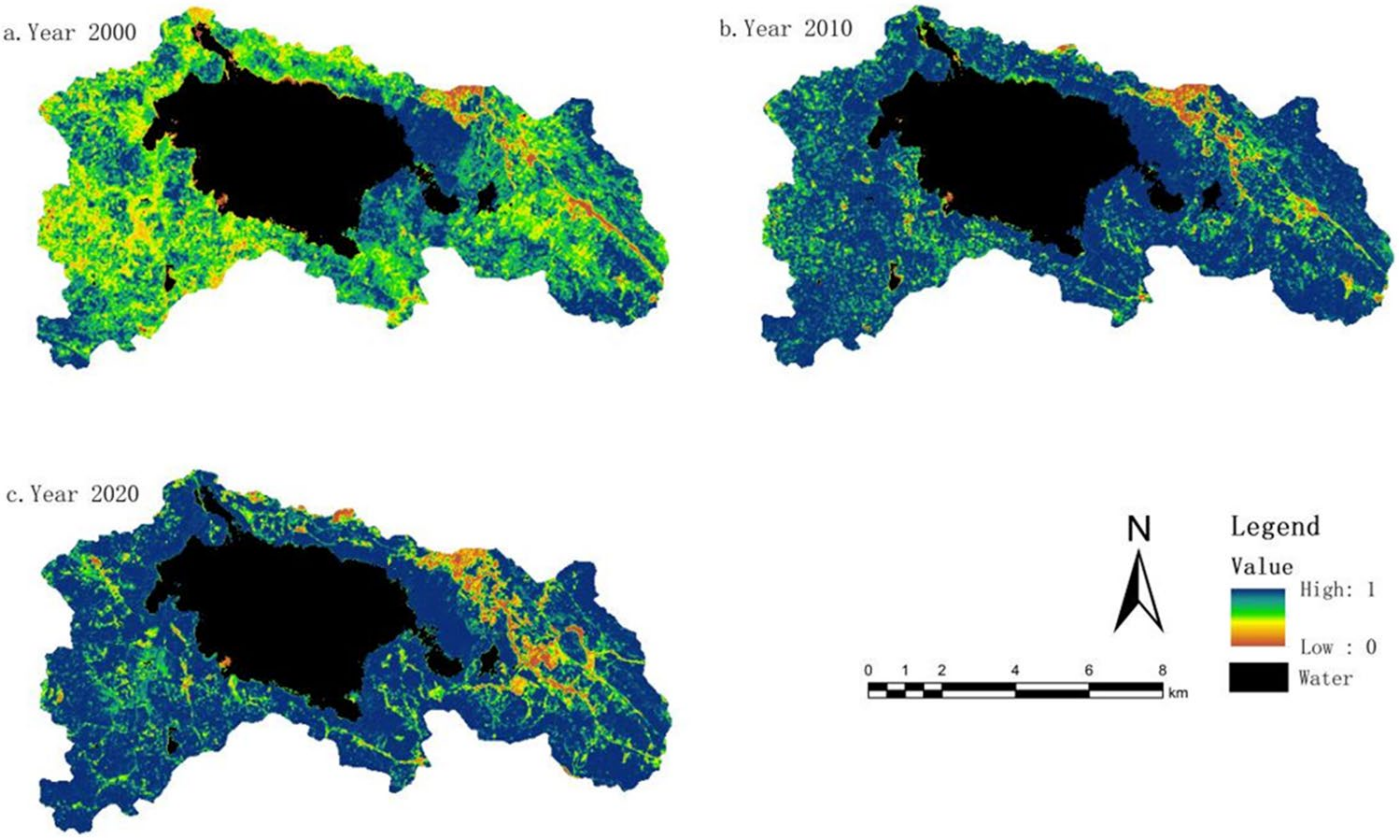
NDVI distribution map from 2000 to 2020. The figure is created using ArcMap 10.7, https://www.arcgis.com.

The NDVI values have been normalized and scaled to a range of 0–1, as shown in Fig. 3. The average NDVI values in 2000, 2010, and 2020 were 0.66, 0.79, and 0.75, respectively. The study indicates an overall trend of initially increasing and then decreasing vegetation greenness. The magnitude of NDVI values is closely related to the surface vegetation coverage in the study area. With the acceleration of urbanization and rapid population growth in the region, the area of land allocated for buildings has continuously expanded, especially with the establishment of the new urban area in Weining County after 2011. This has resulted in a significant reduction in grassland and cultivated land areas. However, based on land use data, it is evident that the vegetation coverage area in the study area has increased from 2000 to 2020. This can be attributed to the implementation of various policies, such as land reforestation and wetland restoration, as well as a series of protective measures within the nature reserve. Overall, the study area has shown a positive development trend in terms of vegetation coverage.

Using ArcGIS software, the NDVI values for different time periods were subjected to differencing analysis. This analysis provided insights into the interannual variations in vegetation greenness, categorized into three types: improvement, no change, and degradation. From 2000 to 2010, the areas showing improvement in vegetation greenness were greater than those experiencing degradation, and the improvement was relatively significant, accounting for 64.9% of the study area. However, from 2010 to 2020, the areas undergoing degradation surpassed those showing improvement, and the largest proportion was areas with no change, accounting for 54.51%. Overall, when considering the entire period from 2000 to 2020, there was substantial improvement in vegetation greenness in the study area. The total area showing improvement exceeded the combined area of no change and degradation, amounting to 55.95%.

#### Wet indicator change characteristics (WET)

The average wet index values for the years 2000, 2010, and 2020 were 0.43, 0.68, and 0.78, respectively. This indicates that the overall wet in the study area has gradually increased with the implementation of relevant conservation policies in the CNRR. However, due to economic and social development, as well as the acceleration of urbanization, the rate of increase in wet has slowed down. Since the Caohai Lake occupies a significant proportion of the study area, the water bodies have some influence on the distribution of wet index. Therefore, the data from each period in the study were masked to exclude the water bodies. The wet index has a positive impact on the ecological environment. Thus, the increase in the wet index represents an improvement in the ecological quality of the study area in terms of wet over the 20-year period.

**Figure 4:**
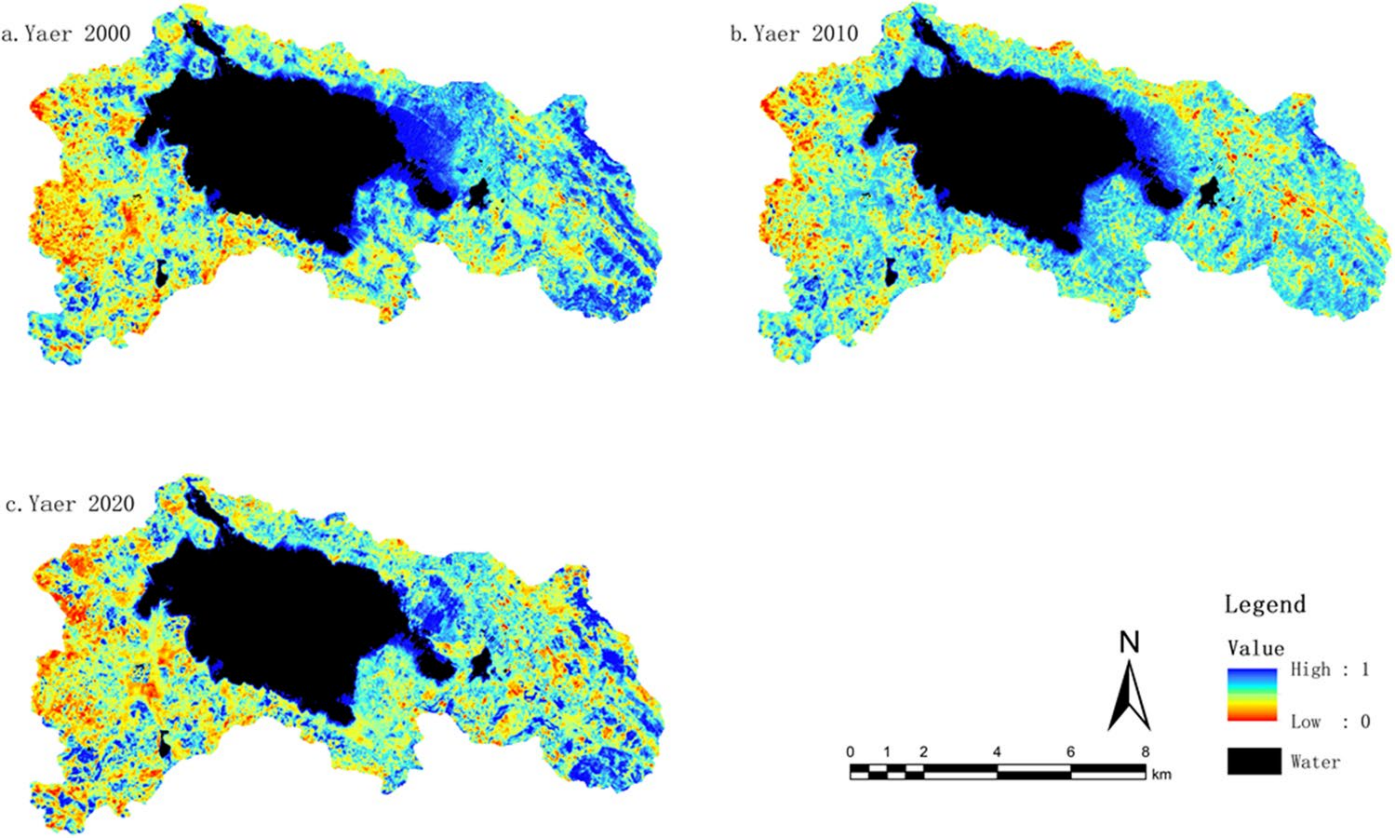
WET distribution map from 2000 to 2020. The figure is created using ArcMap 10.7, https://www.arcgis.com.

By performing differencing calculations on the WET values for different time periods, we obtained the interannual variations in the wet index. From the interannual variations in the wet index (Fig. 4), the improvement in the period from 2000 to 2010 was significantly greater than the degradation, accounting for 89.45% of the study area. From 2010 to 2020, the areas showing improvement exceeded those experiencing degradation, with the largest proportion being areas with stable conditions, accounting for 71.26%. Overall, the study area experienced comprehensive improvement in wet over the period from 2000 to 2020, reaching 96.37% of the total area.

#### Dryness indicator change characteristics (NDBSI)

The normalized difference bare soil index (NDBSI) is closely related to the presence of built-up areas and exposed bare soil. Generally, areas with high building density and exposed soil have higher dryness indices compared to regions with more abundant vegetation growth. The average NDBSI values for the years 2000, 2010, and 2020 were 0.65, 0.59, and 0.64, respectively. By performing differencing calculations on the NDBSI values for different time periods, we obtained the interannual variations in the dryness index. As dryness has a negative impact on the ecological environment, an increase indicates environmental degradation, while a decrease represents environmental improvement.

**Figure 5:**
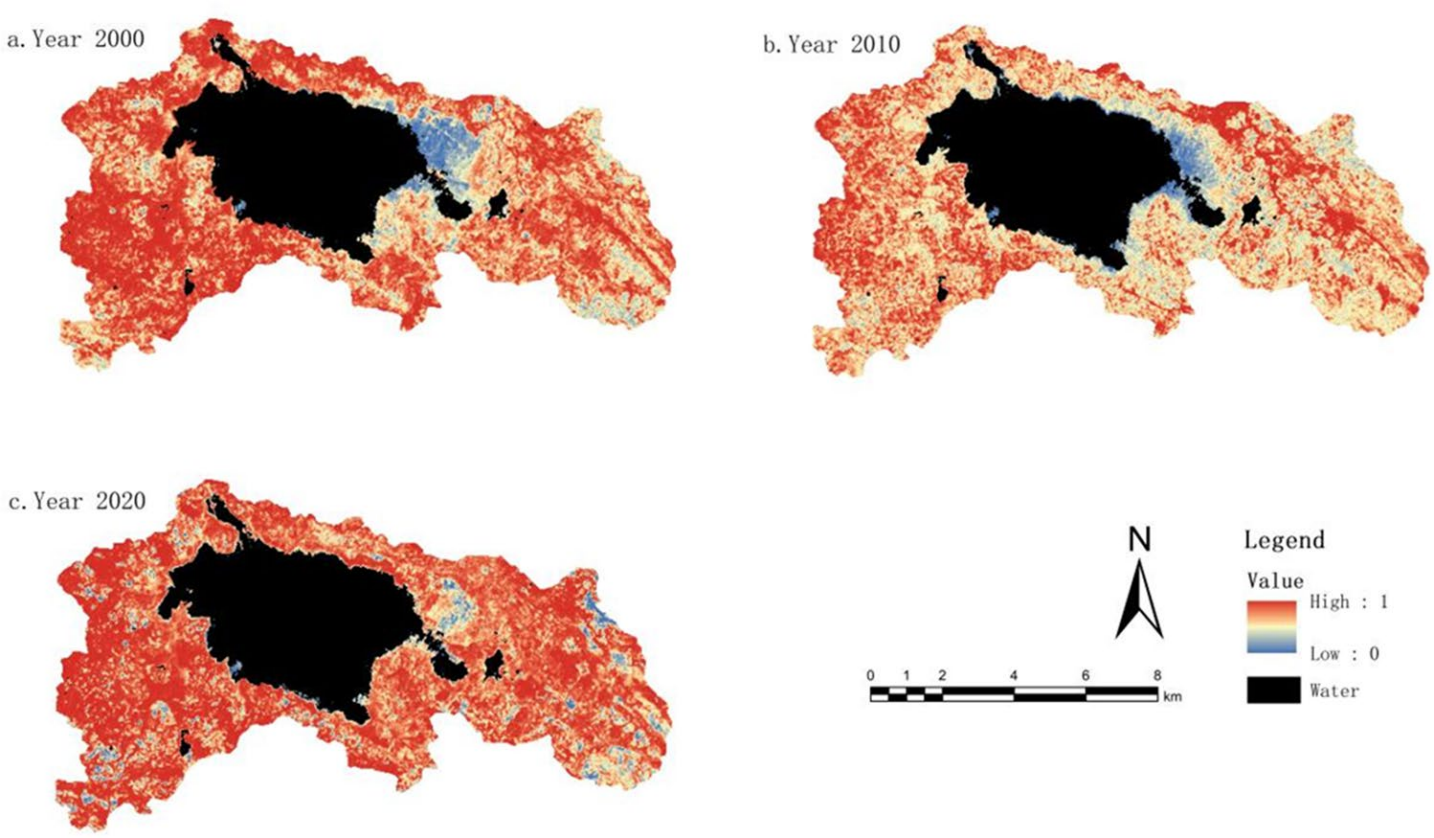
NDBSI distribution map from 2000 to 2020. The figure is created using ArcMap 10.7, https://www.arcgis.com.

From the interannual variations in the dryness index (Fig. 5), the area with a decrease in the dryness index from 2000 to 2010 was larger than the area with an increase, accounting for 43.96%. This suggests that the ecological environment in the region experienced some improvement. However, from 2010 to 2020, the decreasing trend in the dryness index slowed down, while the increasing trend significantly intensified. The area with a decrease in the dryness index reduced to 10.92%, while the area with an increase rose to 46.19%. This indicates that the ecological environment was influenced to some extent. Overall, when considering the entire period from 2000 to 2020, the areas with a decrease, no change, and increase in the dryness index accounted for 43.34%, 33.79%, and 31.98%, respectively. The dominant trend was a decrease, indicating a positive development in the ecological environment.

#### Heat indicator change characteristics (LST)

The average heat index values in the Grassland Lake Protection Area for the years 2000, 2010, and 2020 were 23.79, 26.66, and 28.28, respectively, indicating an overall upward trend in heat intensity. Land surface temperature (LST) primarily refers to the temperature of the ground, which can differ from the commonly referred to air temperature. As heat intensity also has a negative impact on the ecological environment, it suggests that over the 20-year period, the region has experienced some negative effects on the ecological environment due to economic and social development and the acceleration of urbanization.

**Figure 6:**
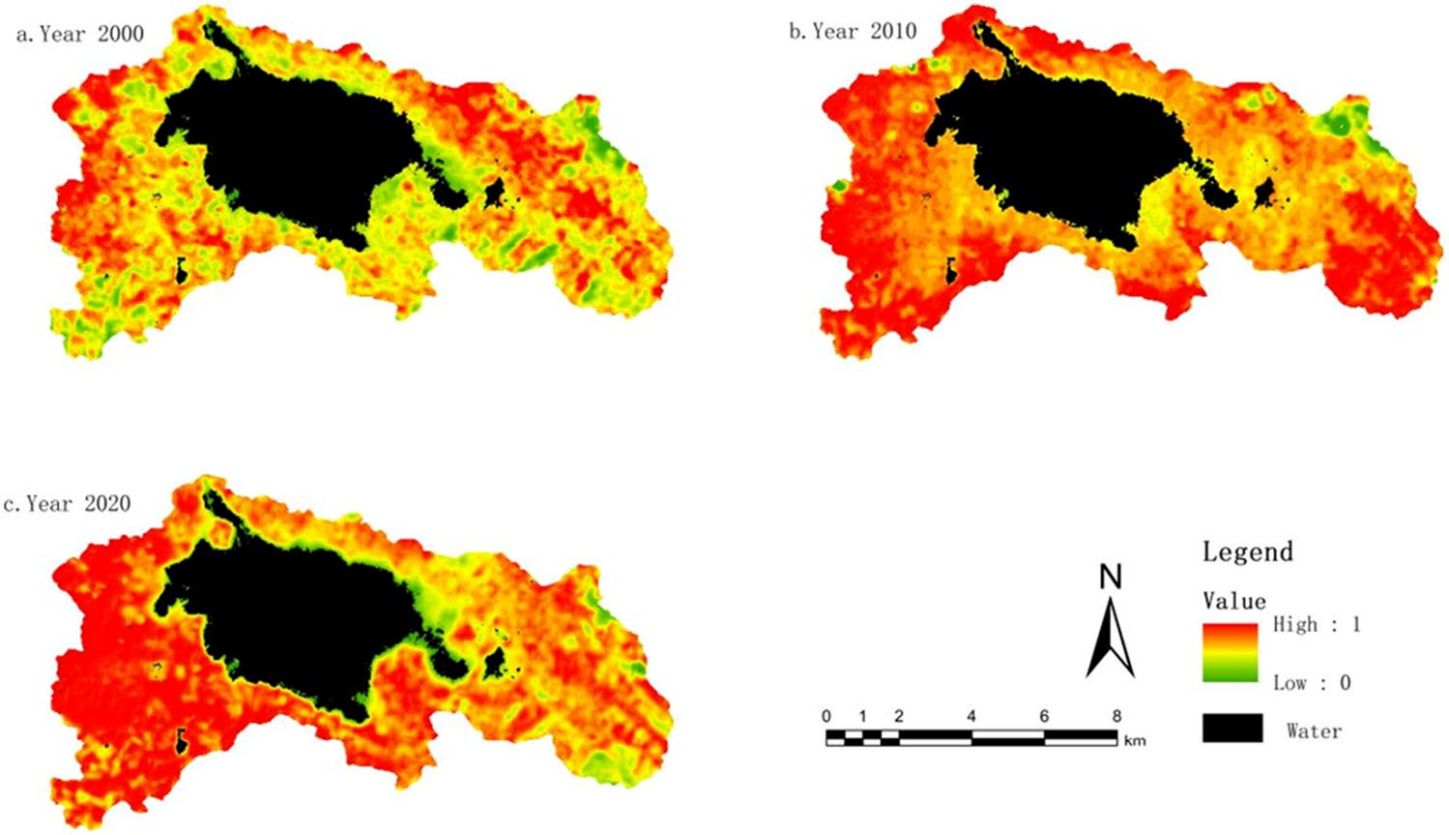
LST distribution map from 2000 to 2020. The figure is created using ArcMap 10.7, https://www.arcgis.com.

By performing differencing calculations on the LST values for different time periods, we obtained the interannual variations in the heat index. From the interannual variations in the heat index (Fig. 6), the proportion of areas with an increase in the heat index was larger than the proportion with a decrease. Specifically, the period from 2000 to 2010 showed a higher increase in the heat index, accounting for 46.32% of the study area. From 2010 to 2020, the rate of increase in the heat index slowed down, but it still accounted for a high proportion of 42.64%. Although the proportion of areas with a decrease in the heat index increased, it remained relatively small compared to the areas with an increase or no change. Overall, when considering the entire period from 2000 to 2020, the proportion of areas with an increase in the heat index exceeded the combined proportion of areas with no change or a decrease. This indicates that the ecological environment in the region is facing certain challenges and requires attention.

#### Temporal and spatial variation characteristics of RSEI

The research utilized ENVI 5.3 remote sensing software to integrate various ecological factors and obtain the RSEI through PCA. According to Table 6, the greenness (NDVI) and wetness (WET) indices for the years 2000 to 2020 are positive values, indicating a positive and beneficial influence on the ecological environment in the study area. On the other hand, the dryness (NDBSI) and heat (LST) indices are negative values, indicating a negative impact on the ecological environment, which hinders the recovery and improvement of the ecological environment in the study area. This result aligns with the actual situation. In the years 2000, 2010, and 2020, the contribution rates of the first principal component were 90.69%, 95.36%, and 91.19%, respectively. In each year, the first principal component had the highest contribution rate among the four components, indicating that the first principal component effectively captured the essential characteristics of each factor to the greatest extent. Therefore, in this study, the original NDVI, WET, NDBSI, and LST indices were replaced by the first principal component to transform them into the new RSEI index, enabling the evaluation of the ecological environment quality in the CNNR.

**Table 6:** Contributions of the four ecological indicators and the RSEI in 2000–2020.

Year	Indicators	PC1	PC2	PC3	PC4	RSEI
2000	NDVI	0.5468	0.3553	0.5380	0.5341	
	WET	0.5087	0.5057	− 0.6734	− 0.1789	
	NDBSI	− 0.4746	− 0.7621	0.2996	0.3229	0.64
	LST	− 0.4659	0.1929	− 0.4091	0.7605	
	Eigenvalues	0.2464	0.0151	0.0050	0.0022	
	Percentage	90.69%	5.62%	2.54%	0.33%	
2010	NDVI	0.5639	0.4695	0.4095	0.5421	
	WET	0.5372	0.3135	− 0.7324	− 0.2771	
	NDBSI	− 0.6016	− 0.7902	0.1123	− 0.0269	0.73
	LST	− 0.5321	− 0.2383	− 0.1774	0.7929	
	Eigenvalues	0.3721	0.0151	0.0021	0.0009	
	Percentage	95.36%	3.86%	0.54%	0	
2020	NDVI	0.4324	0.5783	0.4933	0.4850	
	WET	0.5303	0.4566	− 0.4791	− 0.5299	
	NDBSI	− 0.3967	− 0.3383	− 0.5770	0.6311	0.72
	LST	− 0.6119	− 0.5880	0.4407	− 0.6311	
	Eigenvalues	0.3162	0.0218	0.0077	0.0026	
	Percentage	91.19%	8.52%	0.27%	0	

**Figure 7:**
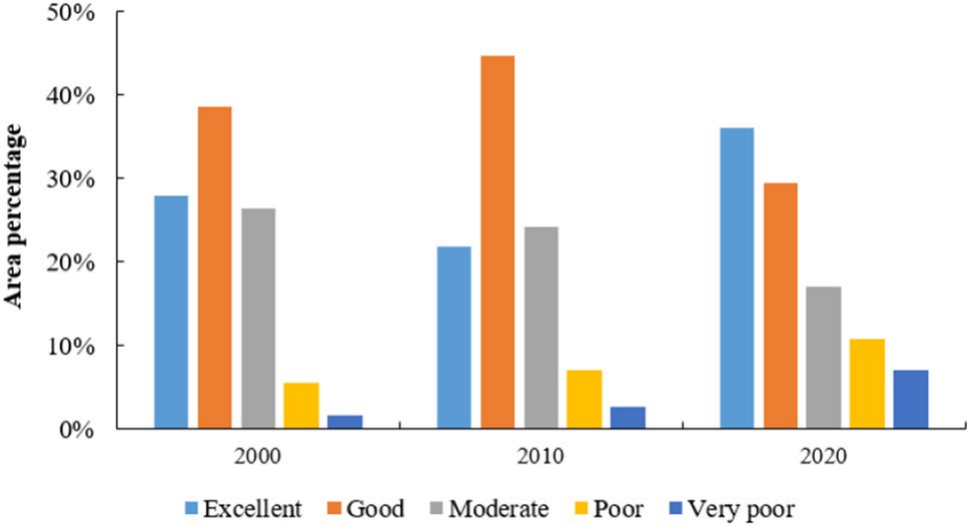
The percentage of RSEI levels in the study area from 2000 to 2020.

To depict the changes and characteristics of the ecological environment in the study area more clearly, and referring to relevant literature, the Remote Sensing Ecological Index (RSEI) has been divided into five levels: very poor (0–0.2), poor (0.2–0.4), moderate (0.4–0.6), good (0.6–0.8), and excellent (0.8–1). As shown in Fig. 7, in the year 2000, the area with poor and very poor ecological environmental quality in the study area was 4.11 km^2^ and 1.14 km^2^, respectively, accounting for 7.05% of the total area. By 2010, this increased to 7.44%, and by 2020, it reached a total of 17.7%. In the year 2000, the area with moderate ecological environmental quality was 19.75 km^2^, representing 26.3% of the total area. By 2010, this decreased to 25%, and by 2020, it further declined to 18%. In the year 2000, the proportion of areas with excellent and good ecological environmental quality was 66.42%. By 2010, the area with excellent and good quality slightly decreased to 66.37%, and by 2020, the proportion of areas with excellent and good ecological environmental quality continued to decline, totaling 65.5%.

**Figure 8:**
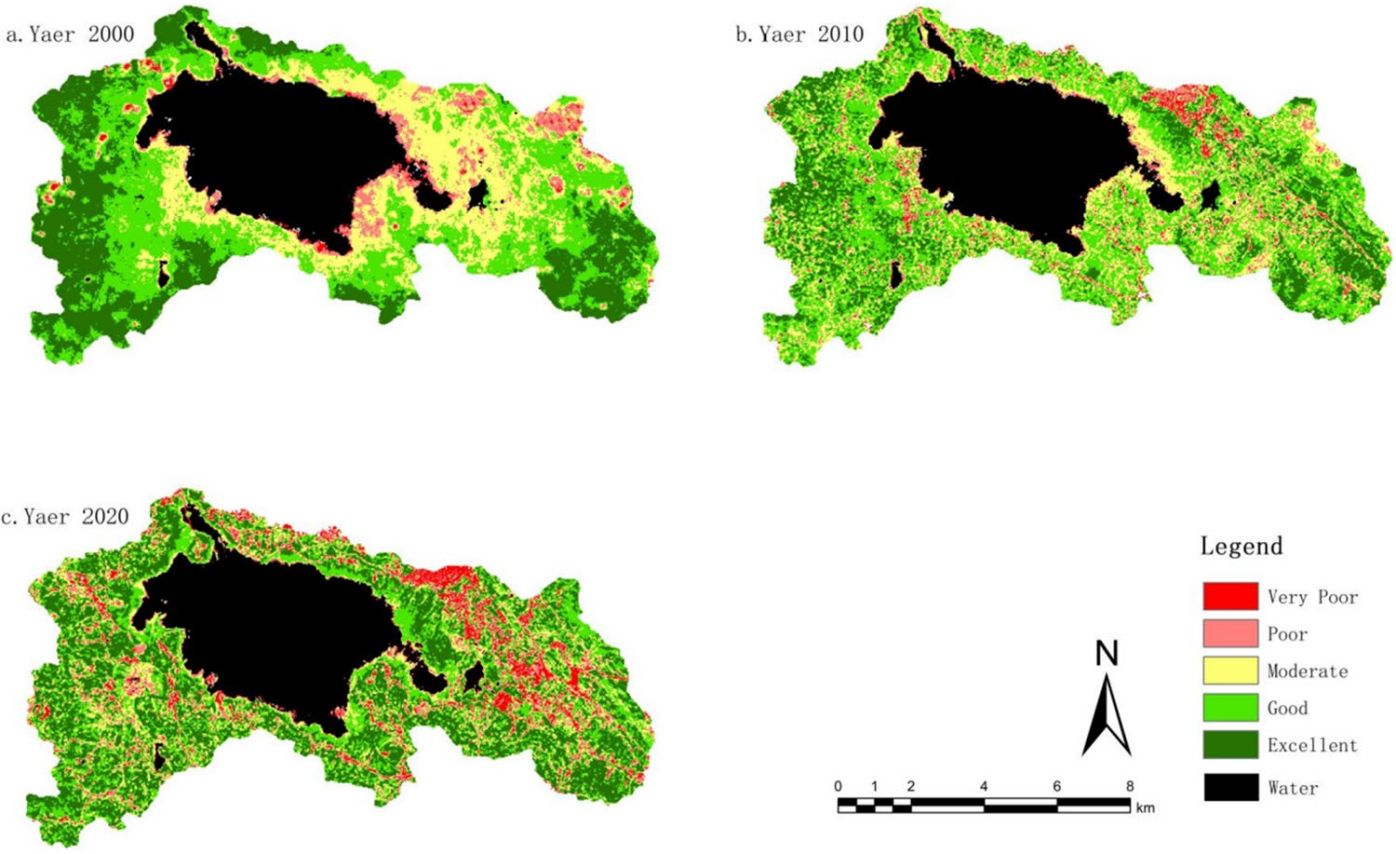
The spatial distribution map of RSEI levels from 2000 to 2020. The figure is created using ArcMap 10.7, https://www.arcgis.com.

From Fig. 8, it is evident that the continuous decline in ecological environmental quality within the CNNR from 2000 to 2020 is primarily concentrated in the Southwest Zone. During this period, land use types have transitioned from grassland to cultivated land and unused land, resulting in a significant deterioration of the ecological environmental quality in this region. Conversely, areas exhibiting a sustained increase in ecological environmental quality are predominantly located in the Caohai Lake Perimeter Zone. In these areas, the conversion of urban and cultivated land into grassland has markedly improved ecological environmental quality. Notably, the Eastern Zone experienced a fluctuation in ecological environmental quality, initially improving and subsequently deteriorating. This region is characterized by high human activity levels, with significant impacts on land use changes. The continuous expansion of construction land has contributed to increased fluctuations and a decrease in ecological environmental quality in this area.

Overall, in the year 2000, areas with “poor” and “very poor” ecological environmental quality were primarily concentrated the Caohai Lake Perimeter Zone and in the eastern part of the CNNR. These regions had high population densities and frequent human activity, resulting in lower ecological environmental quality. The “moderate” level was mainly found in the cultivated and grassland areas around the Caohai Lake Perimeter Zone, while the “excellent” and “good” quality areas were predominantly in regions with lower human activity, such as grasslands and forest areas.

By 2010, a notable transformation was observed, with a considerable portion of areas previously classified as “moderate” shifting to “good” quality, and a smaller portion achieving an “excellent” rating. Furthermore, the categories of “poor” and “very poor” quality, prevalent in the past, showed a clear shift toward the “moderate” level. This indicates an overall improvement in ecological environmental quality within the CNNR, reflecting the positive effects of ecological conservation efforts. However, in areas characterized by frequent human activity, such as urban centers and transportation corridors, the expansion of “poor” and “very poor” quality areas was evident.

By 2020, a significant portion of areas previously rated as “good” and “moderate” had transitioned to the “excellent” category, while some areas regressed to “poor” and “very poor” quality. Although the proportion of “excellent” areas increased, particularly within the core ecological zone around Caohai, the expansion of new urban developments led to an increase in “poor” and “very poor” quality areas.

## Discussion

### Policy implementation

The Southwest Zone of the CNNR is primarily characterized by grasslands, interspersed with pockets of cultivated and forested land. This region has experienced minimal human interference, and its land types have remained relatively stable with minimal fluctuations. However, in recent years, there has been an increasing trend in construction land, leading to a gradual rise in human activities that pose a potential threat to the ecological environment. Consequently, it is imperative for the local government and management authorities to prioritize preventative control measures. Stringent enforcement against illegal construction and land cultivation activities is necessary to preserve the natural ecological succession in this area.

In the Caohai Lake Perimeter Zone, the dominant land types are grasslands and cultivated land, with frequent transitions between grassland and cultivated land during different time periods. From 2000 to 2010, a significant portion of grasslands was converted into cultivated land. Subsequently, from 2010 to 2020, the implementation of the grassland conversion policy led to a substantial transformation, with cultivated land converting into grassland. Human activities exert a considerable influence on the ecological environment of the Caohai Lake Perimeter Zone, resulting in an initial deterioration followed by subsequent improvement in ecological environmental quality. Given its status as the core region of the CNNR, the Caohai Lake Perimeter Zone demands the strictest management and protection measures to prevent recurrent oscillations in ecological quality.

The Eastern Zone of the CNNR primarily consists of grasslands and construction land. This region experiences frequent economic and social activities, despite certain control measures such as the establishment of buffer zones to separate the reserve from urban areas. Unfortunately, these measures have proven less effective, and the ecological environment quality in the Eastern Zone is showing signs of further degradation. Therefore, the Eastern Zone necessitates heightened management and attention, potentially benefiting from artificial ecological restoration methods to rejuvenate the natural ecological environment.

### Limitations and future work

Although our method has shown its efficiency for historical spatial–temporal changes in ecological environment quality assessment, some limitations will be further examined in the future study. For example, due to the calculation characteristics of PCA, water element information needs to be eliminated when calculating RSEI, resulting in the failure to fully consider the ecological benefits of water bodies on the surrounding environment in the ecological environment assessment of the CNNR. The ecological environment status is the feedback effect of a group of environmental factors in space. The lack of any factor (especially water) can lead to inaccurate simulations of ecological conditions. Hence, the appropriate inclusion of aquatic elements in RSEI calculations, particularly in regions predominantly composed of wetlands, is a prospective avenue for future research. Furthermore, there is a need for further investigation into the demarcation and criteria for different zones within the CNNR, as well as the definition of protection goals and standards for each specific area.

## Conclusions

In brief, our analysis of land use changes and eco-environment quality in the CNNR from 2000 to 2020 yielded the following key findings:

1. *Land use trends* Land use changes occurred in two phases: a shift from cultivated land to wetland and a subsequent expansion of constructed land.
2. *Ecological environment quality* Ecological quality improved from 2000 to 2010, stabilizing from 2010 to 2020. Variations in ecological quality were observed across different land types.
3. *Impact of land use change on ecological environment quality* Reverting cultivated land to wetland improved ecological quality, while converting it to constructed land led to degradation.

In conclusion, our study provides valuable insights into land use changes and their ecological environment consequences in the CNNR. These findings have significant implications for promoting the CNNR’s sustainable development, encompassing social, economic, and ecological aspects.

## Data Availability

The datasets used or analyzed during the current study are available from the corresponding author on reasonable request.
